# Dynamic Social Adaptation of Motion-Related Neurons in Primate Parietal Cortex

**DOI:** 10.1371/journal.pone.0000397

**Published:** 2007-04-25

**Authors:** Naotaka Fujii, Sayaka Hihara, Atsushi Iriki

**Affiliations:** Laboratory for Symbolic Cognitive Development, RIKEN Brain Science Institute, Wako, Japan; National Insitutes of Health, United States of America

## Abstract

Social brain function, which allows us to adapt our behavior to social context, is poorly understood at the single-cell level due largely to technical limitations. But the questions involved are vital: How do neurons recognize and modulate their activity in response to social context? To probe the mechanisms involved, we developed a novel recording technique, called multi-dimensional recording, and applied it simultaneously in the left parietal cortices of two monkeys while they shared a common social space. When the monkeys sat near each other but did not interact, each monkey's parietal activity showed robust response preference to action by his own right arm and almost no response to action by the other's arm. But the preference was broken if social conflict emerged between the monkeys—specifically, if both were able to reach for the same food item placed on the table between them. Under these circumstances, parietal neurons started to show complex combinatorial responses to motion of self and other. Parietal cortex adapted its response properties in the social context by discarding and recruiting different neural populations. Our results suggest that parietal neurons can recognize social events in the environment linked with current social context and form part of a larger social brain network.

## Introduction

The complexity of human social organization dwarfs that of any other species. This complexity often results in a heavy cognitive load on our brains, as we are expected to behave in a socially correct manner. The coordination of internal demands and social rules has been called the social brain function [1], [2]. Human social brain function, while adapted to unique evolutionary heights, surely shares many mechanisms in common with that of monkeys, who are therefore ideal subjects in which to study social brain function. But various technical difficulties have made the study of social brain function extremely difficult. Social brain function is tightly linked to social context, and social context consists of multimodal social properties including the behaviors of individuals and details in the environment. Social context changes continuously and is often unpredictable. An action that was socially appropriate a few seconds ago is not guaranteed to be appropriate now. Therefore, if social conflict is to be avoided, frequent updates of each agent's internal representation of the social environment must be an essential brain function. Social brain function tracks current social state and can choose the best solution at the moment. To probe the mechanisms behind social brain function, we must monitor and control a huge number of environmental parameters together with neural activity. Since conventional methods could not handle such massive data, to date there has been almost no study of social brain function at the single-cell level. To solve the technical problem we developed the multi-dimensional recording (MDR) technique [3], which combines of a motion capture system and chronic multi-electrode recording techniques. We used MDR to simultaneously record behavior and parietal neuron activity in two monkeys, M1 and M2, acting in a shared social space. The parietal cortex is thought to contribute to spatial and movement-related cognition [4], [5]. Our aim was to investigate how parietal neurons recognize the actions of self and other, and how they modulate their action-recognition response properties in situations of social conflict arising from unequal social rank.

## Materials and Methods

### Subjects and preparation

**Figure 1:**
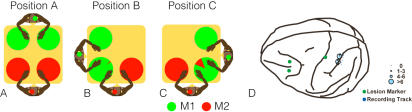
Schematic top views of the task environment for positions A–C, and recording map. During these tasks, we put two monkeys in one of three relative positions (A, B and C) around a table. Each monkey is identified by the color of the circle on his head (green, M1; red, M2). On each trial, we placed a food item at one of four locations in position A and one of three locations in position B and C, indicated by circles on the table. Each circle on the table is a pie chart that depicts each monkey's success ratio for food retrieval in that table location. D. Recording tracks are indicated by blue dots on the brain. The size of each circle centered on the track indicates the number of MR neurons recorded from the track. Green dots indicate the locations of lesion markers for reconstruction.

Two male Japanese macaque monkeys (Macaca fuscata), here called M1 and M2, were used. All procedures were approved in advance by the RIKEN Animal Committee (H18-2B012). A recording chamber was surgically implanted in the left hemisphere of each monkey. We chronically implanted twelve tungsten electrodes (FHC: impedance 800 K–1 M ohm), aiming to record neural activity in an area anterior to the intra-parietal sulcus (IPS) [6]. Most of the motion-related neurons from which we recorded were located in the anterior/medial wall of the IPS (Figure 1D), and were identified by MRI images taken before the experiment. Neuronal activity was recorded by the Digital Lynx system (Neuralynx, Tucson, AZ) and subsequently sorted individually by manual parameters with the Offline Sorter (Plexon, Dallas, TX). Before starting neural recording, we tested the neurons' somatosensory responses. Most of them showed somatosensory responses to right, but occasionally on left, palm, distal and proximal arm, and shoulder. We monitored arm and head movements with a Vicon motion capture system (Vicon Peak, Oxford, UK), sampling at 120 Hz. Monkeys wore motion capture suits which were custom ordered for each monkey. Ten reflective markers (bilateral shoulder, elbow, wrist and hand, as well as forehead and back of head) were attached to each motion capture suit and their locations were reconstructed in three dimensions by the motion capture system. Eight video cameras recorded the entire experimental environment. At the beginning of each recording session we adjusted each electrode's position to obtain the best signal-to-noise ratio, but did not reposition the electrodes during the sessions. During each recording session, neuronal activity was stable and the monkeys' free behavior did not contaminate the neural data with artifact noise. Neural data were collected from nine recording sessions.

### Task

Each monkey was seated in a primate chair that restrained the neck with a collar and the lower half of the body with a plastic cover, but left the upper half of the body, including the arms and head, free to move. The two monkeys were placed around a square table (20″×20″) in one of three different relative positions (Figure 1). The monkeys' relative positions were altered occasionally by the experimenter during the task, introducing social conflict in some positions. Seating the monkeys next to each other (positions B and C) produced potential conflict, since both could reach for a food item placed at the shared corner of the table. Seating them at opposite ends of the table (position A), in contrast, presented no conflict. However, we could reintroduce conflict between the monkeys without changing the seating arrangement in position A by giving the monkeys rakes [6]. When both monkeys had rakes (position A1), conflict ensued because both could reach food placed at the center of the table. When only M2 had a rake (position A2), there was no conflict.

In each trial, the experimenter placed a piece of food on the table so that a monkey could take it. The location of placement was randomly chosen from among four potential locations in position A and three potential locations in positions B and C (locations are indicated by circles in Figure 1). Only one monkey could take the food, since only one food item was placed on the table in each trial. For each relative position, the monkeys performed the task for the 30–40 trials.

### Motion analysis

Motion capture data were analyzed to extract motion episodes. The three-dimensional position of each wrist marker was reconstructed and converted into a single-dimensional trace calculated based on the marker's velocity over time. If the velocity trace of one wrist marker exceeded 30 mm/sec over some period (a motion episode) while all of the other markers remained below 30 mm/sec, we treated the epoch as the exclusive motion episode of that marker. For instance, if the velocity of the marker attached to M1's right wrist exceeded 30 mm/sec in some period during which none of the other markers (including M2's markers) exceed the same threshold velocity, we defined the period as an M1 right arm exclusive motion episode. We did the same analysis separately for each marker. We also defined control periods as epochs in which none of the velocity traces of all four wrist markers exceeded the threshold. The threshold was arbitrarily set at 30 mm/sec through comparison against the noise level of the motion capture system. At this threshold level, we confirmed that we could detect most of visible motions captured in our video recordings.

### Neural analysis

Neuronal activity was analyzed by comparing activity during each of the four exclusive motion episodes and during the control periods. If neuronal activity during an exclusive motion episode was significantly higher than during a control period in position A (Wilcoxon test, *p*<0.05), we classified the cell as a motion-related (MR) neuron. The analysis was done separately for each neuron, for each exclusive episode and for each relative position. After the analysis, each neuron was tagged with four independent binary motion factors including motion of left and right of self and other. Using these four factors, neurons were classified into 16 ( = 2^4^) categories.

Because these categories did not provide realistic social information, we applied two indices to categorize MR neurons. One was the Actor Index and the other was the Action Index. The Actor index has three categories, “Self”, “Other” and “nsp”. “Self” neurons showed an MR response only to own-motion but no response to movements of the other monkey. “Other” neurons only responded to movements of the other monkey. “nsp” neurons responded to motions without monkey specificity. The Action Index was similar to Actor Index, with three categories, “Right”, “Left” and “nsp”. “Right” neurons only responded to right-hand motion regardless of actor. “Left” neurons responded only to left-hand motion. “nsp” neurons responded to motions without hand specificity. Using these two indices, MR neurons were categorized into nine groups.

## Results

### Neural database and adaptive social behaviors of monkeys

We recorded neuronal activity simultaneously from the left parietal cortices of two monkeys (M1 and M2: see materials and methods). 174 neurons were isolated and analyzed. Monkeys sat during recording sessions in primate chairs. We placed the monkeys in three relative positions (Figure 1) around a square table. In position A, they faced each other from opposite sides the table and their reachable spaces did not overlap (Figures 1A). In positions B and C, we placed the monkeys at adjacent edges of the table so that their reachable spaces partly overlapped at one corner (Figures 1B, 1C). On each trial, the experimenter placed a small piece of food on the table. There was no cue indicating which monkey should take the food; each monkey was free to reach for the food or refrain from doing so.

In position A, we placed food at four different locations on the table (Figure 1A: red and green circles). Both monkeys succeeded in taking the food in every trial if the food was within reach. Although they faced each other, each tended to behave as though the other monkey were not present. There was no apparent social interaction or conflict between the monkeys. In position B, the success ratio was significantly biased. If we placed the food where only one monkey could reach it (upper left and lower right in Figure 1B), the monkey who could reach the food took it without hesitation. But when we put the food at the corner (lower left in Figure 1B) where both monkeys could reach it, M2 showed almost no inclination to reach for it. In this space, M1 took the food in 97% of trials. M1 still ignored M2 in this position, but now M2 surreptitiously watched M1. In this position, M2 was aware of M1 and suppressed left-handed action, but M1 continued to behave as though he were alone. Bidirectional social interaction was not yet established between the monkeys. But in position C (Figure 1C), their behaviors were slightly different. In the spaces where only one monkey could reach the food (upper right and lower left in Figure 1C), there was no conflict and no interaction. But in the conflict area (lower right corner in Figure 1C), M2 showed a significantly higher success rate (13%, *t*-test, *p*<0.05) in taking the food than he had shown in position B. On these trials M1 looked at M2 frequently and often threatened M2, especially when M1 lost the trial. At the same time, M2 was peeping at M1's behavior very carefully, looking for tiny chance windows to seize the food for himself. From these behavioral observations, we concluded that M1 was dominant and M2 was submissive. However, we do not know why such asymmetric behaviors occurred between positions B and C. We suspect it was because both monkeys were right handed, giving the advantage to M1 in position B and to M2 in position C – a pattern that M2 must have gleaned from experience during the early trials. The social behaviors described above were consistent over time even though we tested these positions in many orders.

### Response patterns of motion related (MR) neurons in three relative positions

**Figure 2:**
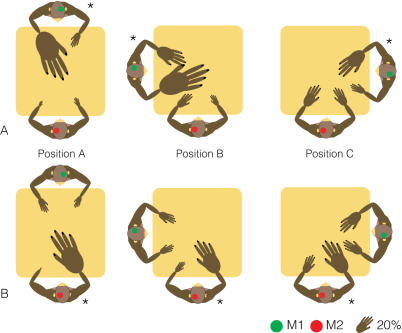
Schematic depiction of the proportion of MR neurons in positions A–C. The size of each hand indicates the percentage of parietal MR neurons that responded to corresponding arm movements. Rows A and B indicate, respectively, the neuronal responses of M1's and M2's MR neurons. The asterisks indicate the monkey from which we recorded activity. Results are separately presented for each relative position. The identification of each monkey is the same as in Figure 1. The size of a hand that corresponds to 20% is shown at lower right.

Our goal was to see how parietal neuron activity correlated with these social behaviors. The first step was motion analysis. From the motion capture data, we extracted four categories of exclusive motion episode—periods in which only one monkey moved one arm. Control periods were similarly extracted by finding periods in which neither monkey moved their arms. These five extracted episode categories (control, M1 right arm, M1 left arm, M2 right arm and M2 left arm) were exclusive and did not overlap in time. From these episodes, we defined neurons as motion-related (MR) if activity during one motion episode was significantly greater than during the control period (Wilcoxon test, *p*<0.05). We performed the same analysis for each relative table position. In our analyses, we exclusively used neuronal activity during control periods in position A to compare MR response in different positions. The responses during four independent motion factors (M1 right arm, M1 left arm, M2 right arm and M2 left arm) for each position characterize the response of each neuron. We found 91 (M1: n = 33 and M2: n = 58) parietal neurons that showed MR response in at least one motion factor in one position. MR neurons were found only in the anterior wall of IPS, where neurons generally showed somatosensory response to right, but occasionally on left, palm, distal arm, proximal arm or shoulder (Figure 1D). Then we counted the number of MR neurons for each motion factor for each position. Proportions of MR neurons are shown schematically in Figure 2 by the relative size of each hand. In position A, 94% of M1's MR neurons (n = 18) and 87% of M2's MR neurons (n = 46) responded to right-hand self movements. In position A, the response patterns of the two monkeys were similar. However, the similarity was broken in position B. M2's neural representation of self-right-hand motion was reduced from 87% to 67%, while M1's neurons maintained the same robust representation of self-right-hand motion (94%). In position C, M2's parietal neurons showed a response pattern similar to that in position B, but M1's parietal neurons reduced the self-right-hand representation from 94% to 60% and increased the representation of other hand motions. The response pattern of M1's parietal neurons again resembled that of M2's neurons in positions C.

**Figure 3:**
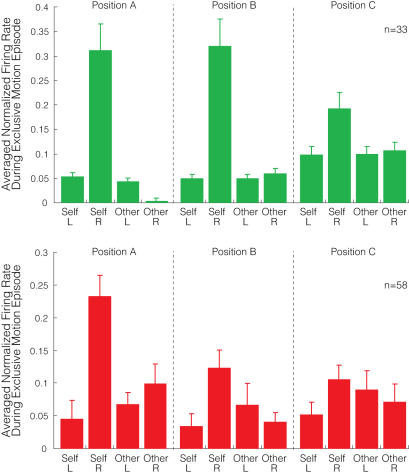
Normalized firing ratio of MR neurons in positions A–C. Bars represent the averaged normalized neural firing rates for four motion factors in three positions. Top (green) represents averaged normalized firing ratio of 33 of M1's MR neurons, and bottom (red) represents 58 of M2's. Error bars indicate standard error.

Figure 2 depicts the cell counts of MR neurons in different table positions, but says nothing about how neuronal firing frequencies were modulated by the same positional switches. We calculated the mean firing rates of 91 MR neurons during exclusive motion episodes and normalized their firing rates by converting them into z-scores using the average and standard deviation of each neuron's activity during a control period in position A. The calculation was performed for each neuron and each motion factor separately. Figure 3 indicates how these neural populations modulated their firing rates in response to positional switches. In positions A and C, response patterns in M1 and M2's neurons were similar, suggesting that sampling bias between the two monkeys was minimal. In both monkeys, the neural firing ratio during self-right-hand action was reduced when the table position was switched from A to C. In position B, there was a discrepancy between M1 and M2's MR responses: M1 kept a robust response to self-right-hand action, but M2's response was reduced. The same modulation tendencies through other position switches were confirmed both in cell counts and firing ratios.

### Actor and Action categorization of MR neurons

**Figure 4:**
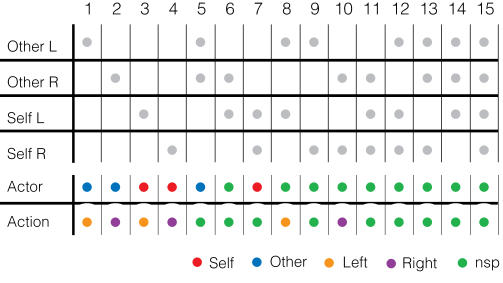
Associations between categories of MR neurons described by four motion factors and categories described by Actor/Action Indices. Using four motion factors, MR neurons were categorized into sixteen groups (of which only fifteen are shown here). Response combinations of the categories are shown in the top table. Numbers shown in the top table indicate group numbers. Each row represents a single motion factor. Grey dots indicate that the group showed positive response to the corresponding motion factor. One group out of the original sixteen that did not show any response has been omitted. The bottom table indicates how Actor/Action Indices describe the fifteen groups above. Each dot's color represents Actor/Action Indices (“Self”, red; “Other”, blue; “Left”, orange; “Right”, purple; and “nsp”, green). For instance, group four only responded to self-right motion, so the Actor and Action Indices of the group were “Self” (red) and “Right” (purple) respectively.

**Figure 5:**
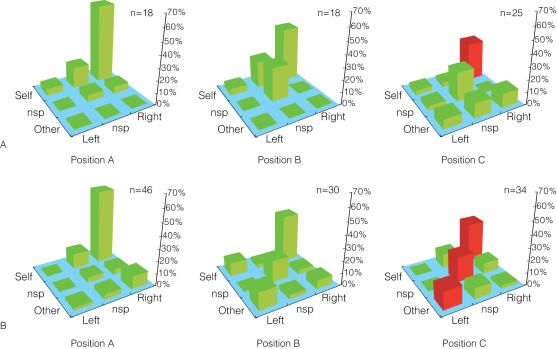
Proportion of MR neurons classified by Actor and Action Indices in positions A–C. M1's and M2's MR neurons (Rows A and B, respectively) were classified into nine groups using the Actor Index and Action Index. Each bar represents the proportions of the corresponding indices. Columns represent positions A–C. Proportions were calculated relative to the total number (shown on top of each graph) of MR neurons in each position. Bars in red indicate statistically significant modulation of that group of neurons in that position compared with the activity of the same group of neurons in position A.

Figures 2 and 3 characterize MR parietal neurons using the four independent motion factors. However, these analyses do not reveal the relationships between the factors. For instance, they do not inform us how neurons responded to one motion that was made in response to other motions. Therefore, we introduced two indices—the Actor Index and the Action Index (see Materials and Methods). The Actor Index tells whose action a neuron responded to (“Self”, “Other” or “nsp”), regardless of the responding arm. The Action Index tells which arm movement neurons responded to (“Right”, “Left” or “nsp”), regardless of the actor. Applying these two indices, we categorized MR neurons into nine groups. Associations between MR response combinations and Actor/Action categories are shown in Figure 4. Figure 5 shows the proportional distribution of the nine groups of MR neurons categorized by these indices. In position A, many of the MR neurons (65% in M1; 61% in M2) exclusively responded to motion of “Self” and “Right” hand. In the analysis used in Figures 2 and 3, we could not calculate significance of modulation because neurons were counted multiple times if they showed positive responses in multiple motion factors. In contrast, in Figure 5, neurons were exclusively categorized into nine groups so that there were no multiple counts across categories. Thus, we compared the proportion of each category in position A with that in position B and then again in position C. (Fisher's exact test, p<0.05) In position B, although the proportion of each category was modulated in a pattern similar what was observed in Figures 2 and 3, no significant difference was detected. However, in position C, we found a significant decrease in the self-right category in both monkeys and a significant increase in the nsp-nsp and other-left categories in M2's MR neurons.

### Spatial and attentional effects on MR modulations

Although MR neural response patterns in both monkeys were similarly modulated when interactive conflict occurred, it was still unclear whether the modulation was induced by social conflict or by other factors, because visual and spatial parameters were also altered by position switches. In an attempt to tease these factors apart, we analyzed MR neuron activity by comparing responses to the motions visible in each animal's left and right hemifields. We found that 24% of parietal MR neurons (22/91) showed a preferred hemifield (Wilcoxon test, p<0.05). Ten neurons preferred motions presented in the right hemi-field and 12 preferred motions in the left hemifield, indicating that there was no significant contralateral spatial preference in left-parietal MR neurons. This suggested that a difference in the visual and spatial parameters between different table positions could be responsible, in part, for the neural modulation data presented above. Parietal cortex is thought to have a role in spatial attention [7]–[9], so these neural responses could be attentional modulations. But this does not rule out a contribution from social factors, because all position switches involved changes both of visual/spatial parameters and of social context.

It is unfortunate we could not track eye positions in this study, but it would have been technically extremely difficult to do this without disturbing the monkeys' natural free behavior. In another attempt to separate MR responses from the potential contribution of spatial attention, we analyzed neural activity in relation to each monkey's head direction, since head movements, especially in naturalistic free-head conditions [10], are closely tied to shifts in spatial attention. However, we could not find a significant head-direction-related response in any of the parietal neurons we had recorded from (Wilcoxon test, p<0.05).

### Application of tool use task and MR modulation

**Figure 6:**
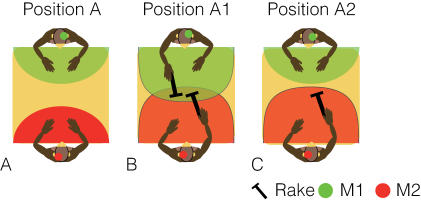
Schematic top views of relative positions and reachable space in positions A, A1 and A2. In all three positions the monkeys were seated at opposite ends of the table. M1 and M2 are discriminated by colored circles (M1, green; M2, red) shown on each head. Each monkey's reachable space is shown on the table by a color-filled curve. Hence, the green area indicates M1's reachable space and the red area indicates that of M2. In position A1, both monkeys were given rake tools; in position A2, only M2 used a rake.

**Figure 7:**
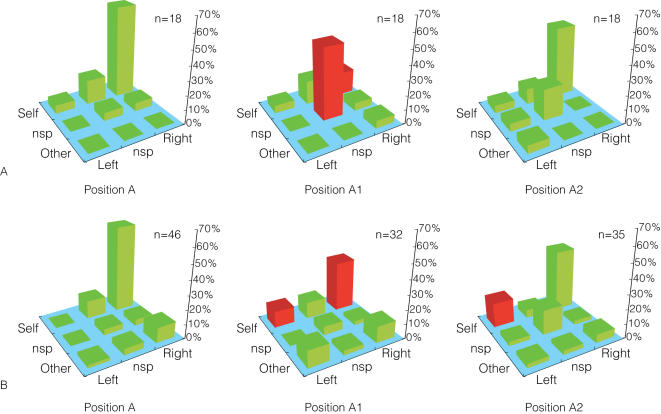
Proportion of MR neurons classified by Actor and Action Indices in positions A, A1 and A2. Figure convention is identical to Figure 5.

In our next attempt, we introduced two new conditions in which the monkeys' relative table positions were identical to position A and social conflict could be manipulated (Figure 6). In position A1, both monkeys were given a rake tool that could be used to obtain food items placed at the center of the table beyond arm's reach. [11] The monkeys already had 2–3 months of training using the rake as a food-gathering tool prior to the recording sessions. Since both monkeys could reach the center of the table, this condition created social conflict. In position A2, M2 was given a rake but M1 was not. Hence there was no overlap between their reachable spaces and hence no conflict. Using these additional conditions we performed the same analysis on the same neural populations shown in Figure 5. Figure 7 indicates the response modulations of MR neurons for positions A1 and A2. As was shown for position C in Figure 5, the proportion of each monkey's self-right MR neurons dropped significantly—from 65% to 17% in M1, and from 61% to 35% in M2 (Fisher's exact test, *p*<0.05). But this time, unlike in position C, the monkeys' viewpoints were nearly identical to position A, and still we observed a marked reduction of self-right neurons in both monkeys. The only big difference this time was the establishment of interactive conflict. When position A2 was applied, in which no conflict occurred, we found that the proportion of self-right neurons was restored in both monkeys to the same levels that had been seen in position A. In positions A1 and A2, neither monkey showed the behavioral suppression observed in M2 in positions B and C.

One concern raised by the introduction of these additional conditions, however, was the effect of rake usage. Rake usage has been shown to induce an extension of parietal neurons' visual receptive fields beyond the hand that wields the tool and onto the rake itself. [11] Conceivably, the introduction of the rake could be contributing to the observed parietal modulation, because the neurons could cover a wider region of space due to wider receptive field. However, there was a suppression of self-right neurons in position A1 and an absence of this suppression in position A2, even though M2 was still using the same tool. Moreover, the neural modulation pattern was similar between positions C and A1. Therefore, we conclude that the effects of receptive field expansion by introducing the rake were independent of, thus have little impact on, parietal neural modulation argued here.

### Dynamic reorganization of MR populations

We observed conflict-dependent modulations of parietal MR neurons, shown in Figures 5 and 7. The robust response properties of parietal neurons to self-right action diminished under conflict situations, and the same population of neurons simultaneously became more responsive to multiple other actions. These findings suggested that a rearrangement of the parietal neural network occurs when a monkey needed to use environmental social information. This raised the question of how these groups of neurons, which responded to motion of self and other under different social circumstances, were dissociated. To address the question, we tracked the neural responses of MR neurons across the various conditions to see when they were preserved. Table 1 shows the result. The table indicates how many parietal neurons responded to four independent actions in positions A, C and A1. It also shows how many neurons preserved the same response properties both in A and C, and both in A and A1. There was a dynamic replacement of the actively responding population whenever conflict existed. For instance, only 23 of the 57 self-right neurons in position A kept their self-right response selectivity in position C, and 15 of the 38 self-right neurons in position C did not show self-right response selectivity in position A. The same tendency was observed in other motions. The proportion of replaced neurons differed from action to action and position to position, but in the larger picture, roughly half of MR neurons active in position A lost their original response properties in positions C and A1, and 40–90% of the MR neurons active in positions C and A1 were new participants. Of course, this leaves a substantial number of neurons overlapping between the various conditions, suggesting that these populations serve common functions across the conditions.

**Table 1:** Cross representation of MR response in position A, C and A1

	MR neuron in position A	MR neuron in position A & C	MR neuron in position C	MR neuron in position A & A1	MR neuron in position A1
Other L	13	6	24	2	14
Other R	15	2	25	4	16
Self L	7	4	19	3	21
Self R	57	23	38	21	34

## Discussion

### Parietal cortex and social cognition

Parietal cortex is thought to play important roles in spatial cognition [12]. It responds to visual [6], [13], auditory [14] and somatosensory [6], [13], [15] stimuli. Because the MR neurons in our study often responded to the motions of the other monkey in the absence of self-motion, it cannot be a pure motor or visual response. Instead, it appears to be generated by an integration of complex cognitive processes [16], including the perceptual extraction of others' bodies and actions from one's visual surrounding [17]–[19], the recognition of a movement's meaning [20]–[23], the differentiation of self from others occupying the same locations in sensory space [24], [25], and the retrieval of knowledge about social hierarchy and judgment rules [26]–[28]. All of these are essential elements in social cognition and are implemented by many brain regions [29]. One of the most famous components of the social brain network is the mirror neuron system. Mirror neurons were found in two cortical sites. One was in the rostral part of ventral premotor cortex (F5). [30] In this area, neurons that encode specific motor actions also responded to passive observation of the same action in the absence of self-movement. The other type of mirror neuron was found in the inferior parietal lobule and was reported to discriminate the intention of an observed action. [31] These mirror neurons have been proposed to play important roles in action cognition and imitation learning. [4], [20], [21]

Both MR neurons and mirror neurons respond to other agents' movements. Mirror neurons respond equally to self-action and action by another, and can discriminate the action's meaning regardless of social context. By contrast, MR neurons responded mostly to self-right arm motion when a monkey was socially isolated but gained responsivity to movement by self and other if placed in a social interaction. The social-context-dependent modulation found in MR neurons has not been reported in mirror neurons. These characteristics of MR neurons suggest that MR neurons are playing different roles from mirror neurons, are involved more in social cognition than in motor cognition, and may share current social information with other relevant cortical and sub-cortical areas [32]–[34].

### Contribution of MR neurons in social cognitive network

Our results show that the parietal cortex modulates its responses to motion events according to social context. When the monkeys were socially detached, even though they sat near each other, each monkey's parietal neurons tended to respond more to his own right-hand motion than to actions of the other. The laterality (preference for responding to stimuli appearing in contralateral space) was robust. This and other neural response properties we observed were consistent with previous studies. [11], [35] However, if conflict of interest between the monkeys was present, parietal neurons lost their marked preference for self-generated movements and began responding to other motion events in two ways. One way was a general arousal-type response (nsp–nsp) to social events regardless of actor and action types. There was almost no nsp–nsp response when the monkeys were socially isolated, but it emerged when the monkeys started sharing a social space. In terms of social cognition, this might be a source of social attention that alerts one to socially important events. The other response type carried specific information about the actor and action properties of motion events in the shared space. Combining these two response types, parietal cortex may provide important social information to the wider social brain network for use in the selection of socially correct behavior.

### Dynamic reorganization of neural responsiveness and adaptive behavior

We found dynamic reorganization of the response properties of parietal neurons when social context was manipulated. Neurons that showed MR responsivity in one condition could immediately lose this responsivity in another condition that had almost identical motor requirements and visual aspects, and in which social context was the only significant variable. In many cases, more than 50% of MR neurons were replaced in response to such a contextual switch. Our findings suggest that in novel social contexts, existing neural populations that are already adapted to a similar context acquire new functional features by recruiting new sets of neurons while discarding others. If this is happening in parietal cortex, we should expect to find the same kind of momentary adaptive neural modulation occurring in other cortical or sub-cortical components of the social brain network. Since social context keeps changing, the social brain network must continuously reform its functional structure. The highly flexible social brain function that stems from this dynamic reorganization is a hallmark of primate and human social complexity, making it a crucial research frontier if we are to understand our evolutionary origins and our uniquely advanced social nature.
